# Transcriptomes That Confer to Plant Defense against Powdery Mildew Disease in *Lagerstroemia indica*

**DOI:** 10.1155/2015/528395

**Published:** 2015-07-12

**Authors:** Xinwang Wang, Weibing Shi, Timothy Rinehart

**Affiliations:** ^1^USDA-ARS, Crop Germplasm Research, College Station, TX 77845, USA; ^2^Texas A&M AgriLife Research and Extension Center, Dallas, TX 75252, USA; ^3^Department of Plant Pathology, Texas A&M University, College Station, TX 77843, USA; ^4^USDA-ARS, Thad Cochran Southern Horticultural Laboratory, Poplarville, MS 39470, USA

## Abstract

Transcriptome analysis was conducted in two popular* Lagerstroemia* cultivars: “Natchez” (NAT), a white flower and powdery mildew resistant interspecific hybrid and “Carolina Beauty” (CAB), a red flower and powdery mildew susceptible* L. indica* cultivar. RNA-seq reads were generated from* Erysiphe australiana* infected leaves and* de novo* assembled. A total of 37,035 unigenes from 224,443 assembled contigs in both genotypes were identified. Approximately 85% of these unigenes have known function. Of them, 475 KEGG genes were found significantly different between the two genotypes. Five of the top ten differentially expressed genes (DEGs) involved in the biosynthesis of secondary metabolites (plant defense) and four in flavonoid biosynthesis pathway (antioxidant activities or flower coloration). Furthermore, 5 of the 12 assembled unigenes in benzoxazinoid biosynthesis and 7 of 11 in flavonoid biosynthesis showed higher transcript abundance in NAT. The relative abundance of transcripts for 16 candidate DEGs (9 from CAB and 7 from NAT) detected by qRT-PCR showed general agreement with the abundances of the assembled transcripts in NAT. This study provided the first transcriptome analyses in* L. indica*. The differential transcript abundance between two genotypes indicates that it is possible to identify candidate genes that are associated with the plant defenses or flower coloration.

## 1. Introduction

The genus* Lagerstroemia* is a deciduous shrub or small tree, originally native to southeastern Asia that is also cultivated and naturalized in temperate and tropical regions worldwide [1]. It has been naturalized in the Southern United States after being introduced in the late 1700s and became one of the most distinctive and popular summer flowering woody ornamentals in United States Department of Agriculture (USDA) Hardiness Zones 6 through 9 from east to west coast [2]. Because it flowers all summer, has attractive exfoliating bark, and is well adapted to various soil types (acidic, alkaline, and saline), it has been called “the lilac of the south” [1]. The US Department of Agriculture Census of Horticultural Specialties [3] concluded that the retail and wholesale sales of crape myrtle account for over $46.5 million annually, and nearly 90% of these are from 11 states (Alabama, California, Florida, Georgia, Louisiana, Maryland, North Carolina, South Carolina, Tennessee, Texas, and Virginia). Of these states, Texas accounts for 17% ($7.7 million) of the total US carpe myrtle sales, making it an important ornamental crop for the economy of the state.

There are 56 [4] to 80 [5] species in the genus* Lagerstroemia*. Only two species,* L. indica* and* L. fauriei*, are popular cultivated ornamental flowering plants in the United States.* Lagerstroemia indica* species has long summer flowering time (up to 120 days) but suffers disease problem, especially powdery mildew in the southern US.* Lagerstroemia fauriei* has a shorter flowering time (~20 days) annually but is highly powdery mildew resistant. Although interspecific hybridization between these two species has resulted in the release of several popular cultivars with improved disease resistance [1, 6], genetic information about the disease-resistance trait and genomic resources to uncover the genetic basis for resistance in this genus are lacking. Until the early 1970s, most crape myrtles grown in the United States were cultivars of* L. indica* (common name crape myrtle). In the past four decades, several disease-tolerant cultivars were introduced including interspecific hybrids between* L. indica* and* L. fauriei* [6]. Additional efforts are still needed to improve plant size and shape, expand the range of flower colors, extend flowering period, enhance bark attractiveness, improve fall foliage color, and increase disease resistance. Newly released cultivars still lack sufficient levels of resistance to mildew disease in humid southern climates despite improvements over older cultivars, which were mostly pure* L. indica* selections.

Powdery mildew is a serious disease of crape myrtle in the USA [7]. When the* L. fauriei* genetic background was introduced in crape myrtle breeding, the problems with this disease were partially overcome by planting interspecific hybrids that are disease resistant [1]. Although the most durable forms of disease resistant germplasm can be found in cultivars that contain* L. fauriei* background, some moderate levels of resistance to powdery mildew have also been observed in the cultivars derived from pure* L. indica* [1, 5, 6, 8]. Extensive breeding efforts have made substantial progress on ornamental traits such as flower and leaf color in* Lagerstroemia* breeding program. However, genetic information and studies on important traits such as disease and insect resistance, drought, and cold hardy tolerance have lagged behind.

At the molecular level, plant response to pathogens involves the activity of two classes of receptors: receptors localizing at the plasma membrane (PM) and receptors that typically recognize conserved microbial motifs referred to as PAMPs/MAMPs (pathogen-/microbe-associated molecular patterns) [9]. Although plant-fungus interaction commences with the contact between the plant and spore surfaces, various genes are involved in plant defense against pathogens [10]. It is a well-known fact that* Arabidopsis* plants avoid penetration by many nonhost fungal pathogens (mainly powdery mildews) as a result of the expression of penetration genes (PEN), providing a quick and efficacious defense response at the cell wall. Plant roots, stems, and leaves are the most sensitive tissues to pathogens infection [11]. Therefore, the gene expression pattern in these tissues during the* E. australiana* infection was examined by looking at transcript abundance.

Genomic-based breeding plays an important role in the identification of genes governing specific traits especially in woody plants [12]. The development and emergence of next generation sequencing (NGS) technologies have driven all biological disciplines in the last few years as documented by the increase in DNA sequencing data and emphasis. NGS technologies create a vast amount of short sequence data, presenting many problems to computational biologists, bioinformaticists, and end-users endeavoring to assemble and analyze NGS data in novel ways. DNA markers such as SSR and SNPs can be easily obtained from these NGS sequences for genetic linkage map development and gene location.

RNA-seq is a NGS method that sequences the transcriptome containing all RNA transcripts. This method uncovers the expressed sequences in specific tissue at a specific time and is rapidly replacing other methods of studying gene expression such as microarrays [13]. It is practical in nonmodel plant species mainly because a reference genome is not required. RNA-seq data can be used to characterize differential expression or tissue-specific transcripts. Ward et al. [14] reviewed approaches for the analysis of short-read transcriptome data for nonmodel species for which the genome of a close relative was used in place of a true reference genome. For those species that do not have a reference genome, a* de novo* assembly method holds more power for discovering unique sequences and provides the possibility of simultaneously querying the transcripts and expression levels in multiple organisms or tissues in a system [15, 16].

Recently, PCR-based molecular tools were successfully used to unambiguously identify different crape myrtle cultivars, compare parentage, and verify interspecific hybridizations [17–21]. To our knowledge, no genetic sequence information and transcriptome information of this species have been available, particularly regarding tissue-specific disease resistant genes. In this study, next generation sequencing technology was used to investigate and compare transcriptomes in powdery mildew resistant and susceptible* L. indica* cultivars, in order to (1) generate leaf-responsive gene expression profiles, (2) identify differentially expressed genes between resistant and susceptible* L. indica* cultivars, and (3) provide useful genome sequences for* Lagerstroemia* molecular breeding and genetics study. The genome transcriptome sequences obtained in this study provide fundamental reference for the discovery of important gene alleles of interests, which are related to flower color, disease resistance, and/or other (a)biotic stresses. In addition, the transcriptome sequences will serve as genetic resources to develop markers (e.g., microsatellites or SSRs) that benefit the marker-assisted selection (MAS) especially in the genomic-based ornamental breeding program.

## 2. Materials and Methods

### 2.1. Sample Preparation

Two* Lagerstroemia* cultivars, “Carolina Beauty” (CAB) and “Natchez” (NAT), were used for the study. CAB has red flowers and is highly susceptible to powdery mildew; NAT is a white flowered interspecific hybrid derived from* L. indica* ×* L. fauriei* and highly resistant to powdery mildew. To observe gene expression related to differing powdery mildew resistance between these two cultivars, natural inoculum sources of* Erysiphe australiana* that were initially considered highly pathogenic in* L. indica* susceptible cultivars were collected from other infected foliage (50 leaves/plant) [22]. The infected leaves were used to inoculate CAB and NAT plants in a temperature-controlled greenhouse (29°C and 70% relative humid) prior to sampling for RNA isolation. Healthy plants were monitored every two days for disease development [8]. Newly opened young leaves with visible epiphytic conidia (CAB, not NAT) at 14 days after inoculation were collected from both genotypes, washed, and dried with paper towel for total RNA isolation.

### 2.2. Total RNA Isolation, cDNA Library Construction, and Sequencing

Total RNA was obtained from young leaves of powdery mildew susceptible (CAB) and resistant (NAT)* L. indica* cultivars using the RNeasy mini kit following the manufacturer's instruction, treated with RNAase-free DNAase, and repurified with the RNeasy kit (Qiagen, Valencia, CA, USA). To enrich the sequence depth of message RNA (mRNA), a poly-A selection method was used to remove the overwhelming ribosomal RNA (rRNA) and transfer RNA (tRNA). The enriched mRNA samples were subjected to standard Illumina cDNA library construction and sequenced using Illumina sequencing platform Hiseq 2000 (100-paired). Briefly, mRNA was first purified using poly-A selection from total RNA, then chemically fragmented, and converted into single-stranded cDNA using random hexamer priming. The second strand was generated to create double-stranded cDNA. cDNA library construction was performed by using TruSeq reagents from Illumina Inc. (San Diego, CA) following the manufacturer's recommendations. Transcriptome sequencing using Illumina HiSeq 2000 was performed at the Center of Genomics and Bioinformatics, Texas A&M University.

### 2.3. Transcriptome Sequence Assembly and Annotation

To remove the low quality nucleotides and potential ribosomal RNA sequence, the raw Illumina reads were trimmed and aligned against the Silver database [23] using a JGI (Joint Genome Institute, United States Department of Energy) developed filtering script. To get the transcriptome reference genome, the artifact-filtered reads from CAB and NAT samples were used for* de novo* assembly using Rnnotator [24], an automated* de novo* RNASeq assembler. To maximize the assembly of each transcriptome, multiple hash values were used for different rounds of velvet assemblies [24]. After velvet assemblies, the resulting contigs were merged through Minumus2, and the duplicated and small contigs that were covered by long contigs were removed using Vmatch (http://www.vmatch.de/) to make the final reference contigs. The initial short reads data sets are available at the NCBI Short Read Archive (SRA) with the accession number SRX212270. The assembled sequences (500 bp and above) can be accessed from NCBI's TSA database with TSA BioProject number PRJNA236373.

To do functional analyses at the gene and pathway levels, the putative open reading frames (ORFs) were predicted using EMBOSS/GETORF software [25]. Unigenes larger than 300 bp were annotated by searching the KEGG database (release 58.1, June 1, 2011) [26] using BLASTX algorithms at an *E*-value cutoff of 1 × 10^−5^.

### 2.4. Digital Expression Analysis

To identify the differentially expressed genes (DEGs) and metabolic pathways between powdery mildew resistant and susceptible* Lagerstroemia* cultivars, the shot-gun short reads data from the leaves of both genotypes were separately mapped against the combined unigene sequence from* de novo* assembly through a Burrows-Wheeler Aligner (BWA) based JGI in-house developed gene counting software at a cutoff of 97% identity [27]. To apply statistical analysis, the mapped reads were converted into a counts matrix table using SAMtools (http://samtools.sourceforge.net/). Then, the raw counts were normalized into reads per million (RPM), which were used to indicate the relative abundance of transcripts [27, 28].

To identify the unigenes that consistently showed significant differences between the resistant and susceptible genotypes, a JGI in-house developed R script that applies multiple statistical methods including DESeq, edgeR, Wilcoxon, and rankprod was used for analysis. The *P* values for multiple tests were corrected using the Benjamini-Hochberg approach [29]. To identify the metabolic pathway potentially associated with the trait of powdery mildew resistance or susceptibility, the pathway enrichment analyses were conducted by using the Fisher's exact test based on the enriched genes in differential expression levels and known KEGG pathways (*n* = 293).

### 2.5. qRT-PCR Analysis to Validate Transcript Abundance Differences

Aliquots of total RNA used for sequencing as described earlier were used for qRT-PCR [30]. cDNA was synthesized and quantified using the Nanodrop (ND-1000) spectrophotometer (Nanodrop products, Wilmington, DE). Twenty-one primer pairs from differential expressed candidate genes were designed using an online PRIMEGENS program (http://primegens.org/) and synthesized from IDT Technologies (Integrated DNA Technologies, Coralville, IA). The working stock of each primer was adjusted to 2 *μ*M. The gene IDs and primer sequences were listed in Table S1 in Supplementary Material available online at http://dx.doi.org/10.1155/2015/528395. Real-time PCR was run on BioRad C-1000 (CFX96) real-time system using the iQ SYBR Green Supermix (BioRad Laboratories, Hercules, CA). For internal control, two housekeeping genes, cab-h1 and cab-h8, from nondifferential expressed genes were used to calculate threshold differences and fold expression differences from differential genes. The abundance levels of the selected transcripts normalized to housekeeping genes were calculated using the 2^−ΔΔCt^ method [31]. Before conducting qRT-PCR, regular PCR was performed at an initial denature at 94°C for 3 min, followed by 94°C 40 sec, 58°C 30 sec, and 72°C 40 sec for 20 cycles with a final extension of 3 min at 72°C.

## 3. Results

### 3.1. Transcriptome Sequencing and* De Novo* Assembly

Total RNA from young leaves of powdery mildew susceptible (CAB) and resistant (NAT)* L. indica* cultivars was isolated. To enrich the sequence depth of message RNA (mRNA), a poly-A selection method was used to remove the overwhelming ribosomal RNA (rRNA) and transfer RNA (tRNA). The enriched mRNA samples were subjected to standard Illumina cDNA library construction and sequenced using Illumina sequencing platform Hiseq 2000 (100-paired). As shown in Table 1, a total of 16,818,312 (1.64 GB) and 17,856,916 (1.74 GB) reads were generated from susceptible and resistant genotype cDNA libraries, respectively. The raw reads were further searched against Silver database [23] to remove the residue of rRNA reads and also the low quality reads, and consequently only ~0.7% of reads were filtered out. As described in the methods, the filtered high quality shot-gun transcriptome reads were subjected to Rnnotator for* de novo* assembly. As part of the pipeline, 48.26% and 47.09% of duplicated reads were removed from high quality reads datasets of susceptible and resistant samples, respectively (Table 1). After cleaning, the pipeline performed the* de novo* assembly using velvet (Version: 1.2.03) with different hash lengths, resulting in 111,804 and 112,639 contigs with a N50 of 1,110 and 1,081 bp for susceptible and resistant samples, respectively (Table 1). In total, 224,443 contigs were assembled in the* L. indica* species. All the assembled contigs and data have been archived at the NCBI Sequence Read Archive (SRA) under BioProject ID PRJNA236373 (http://www.ncbi.nlm.nih.gov/bioproject/236373).

### 3.2. Functional Annotation of Contigs/Isotigs

**Figure 1:**
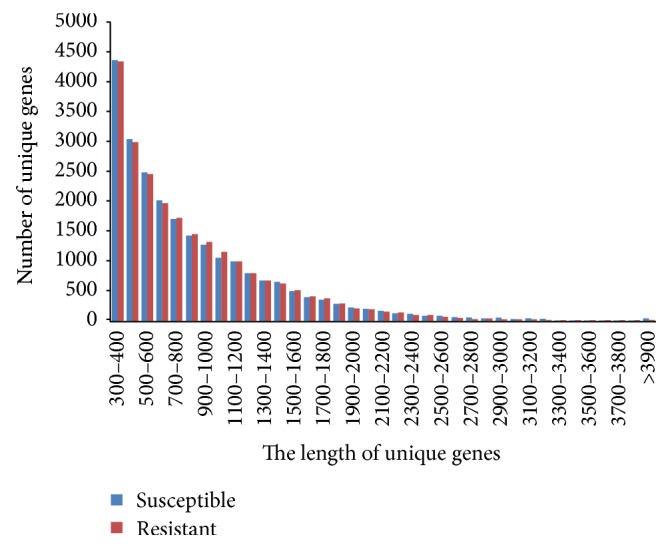
Length distribution of unigenes in the assembled transcriptomes of two* Lagerstroemia indica* cultivars: powdery mildew susceptible (Carolina Beauty, CAB) and powdery mildew resistant (Natchez, NAT).

For functional annotation of assembled contigs, the open reading frames (ORFs) were predicted and the coding genes were removed using the clustering function of Vmatch (http://www.vmatch.de/), resulting in 23,564 and 23,387 nonredundant coding genes for susceptible and resistant cultivars, with mean length of the unique genes of 878 and 860 bp, respectively (Table 1). The nonredundant coding genes in the two genotypes were identified and a total of 37,035 unigenes were found in* L. indica* species. After annotation, there are 20,118 out of 23,564 and 19,898 out of 23,387 predicted genes have function annotated by KEGG database for CAB and NAT cultivars. In total, approximately 85% of predicted genes with known function have been annotated (Table 1). The length distribution of the predicted unique genes in the sequenced two genotypes also had very similar patterns, which suggests no bias was introduced during the construction of the cDNA libraries (Figure 1).

### 3.3. Differentially Expressed KEGG Genes Related to Response to Powdery Mildew in* L. indica* Species

**Figure 2:**
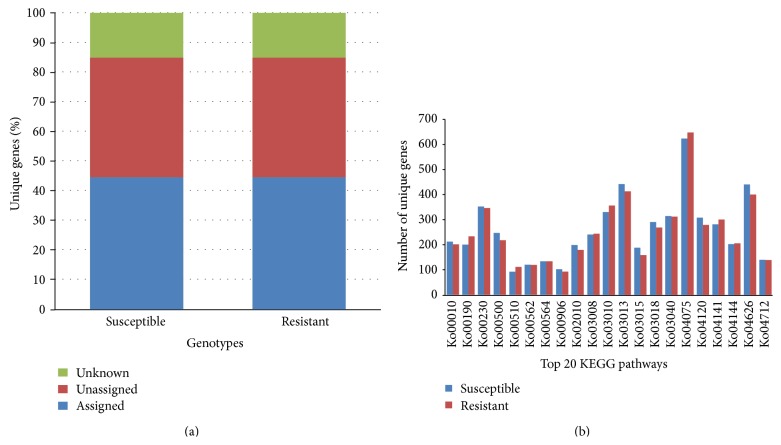
Distribution of active genes in two* Lagerstroemia indica* cultivars: powdery mildew susceptible (“Carolina Beauty,” CAB) and powdery mildew resistant (“Natchez,” NAT) transcriptomes. (a) KEGG database based gene annotation. (b) Top 20 active KEGG pathways based on the number of unigenes.

To explore the possibility that changes in gene expression might be responsible for the differences between the resistant and susceptible cultivars, the coding gene sequences were combined from assembled contigs of both cultivars and shown to have similar genetic background, gene structure, and distribution (Table 1, Figures 1 and 2). Then, the redundant or duplicated genes were removed resulting in a total of 224,443 unigenes for further study (Table 1).

**Figure 3:**
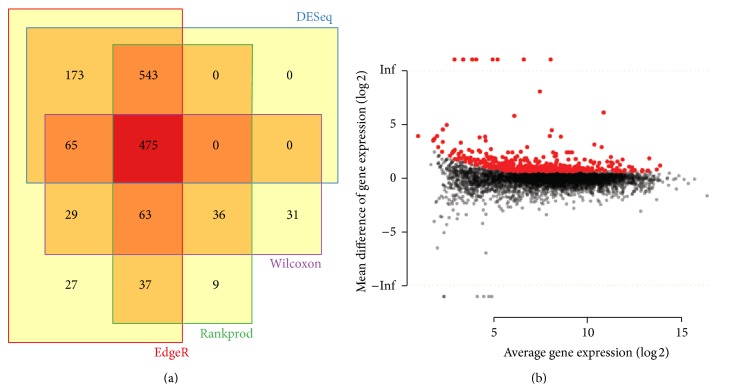
Differentially expressed genes (DEGs) identified by different statistical methods (a) and global review of differentially expressed genes in powdery mildew resistant (red dot) and susceptible (black dot) genotypes in* Lagerstroemia indica* species (b).

Read counts were grouped by KEGG IDs to identify alleles of unigenes that may play the same role in metabolic functions. The normalized KEGG gene expression matrix table was subjected to multiple statistical calculations to identify differentially expressed genes (DEGs). As shown in Figure 3, there are a total of 1,412, 1,256, 1,154, and 699 KEGG genes differentially expressed based on four statistical methods: edgR, DESeq, rankprod, and Wilcoxon, respectively. To reduce the bias of different statistical methods and identify the conserved sets of unigenes that are associated with the resistant or susceptible phenotype, the read counts were normalized by reads per million (RPM). Notably, there are a total of 475 KEGG genes that show significant difference in all tested statistical methods (Figure 3). More interestingly, there are five (K13230, K05280, K00660, K00487, and K08081) out of top 10 differentially expressed KEGG genes involved in the biosynthesis of secondary metabolites (Table 2). Among these differentially expressed KEGG genes, 2,4,7-trihydroxy-1,4-benzoxazin-3-one-glucoside 7-O-methyltransferase (K13230), a gene encoding enzymes [EC:2.1.1.241] involved in the last step of benzoxazinoid biosynthesis (ko00402) to produce DIMBOA-glucoside, has 67.7-fold abundance change in the resistant genotype compared to the susceptible genotype (Table 2). In addition, four out of top 10 KEGG genes (K05280, K00660, K00487, and K05279) came from the flavonoid biosynthesis pathway (ko00941) (Table 2).

### 3.4. Pathway Enrichment Analysis for the Unique Gene Expression

**Figure 4:**
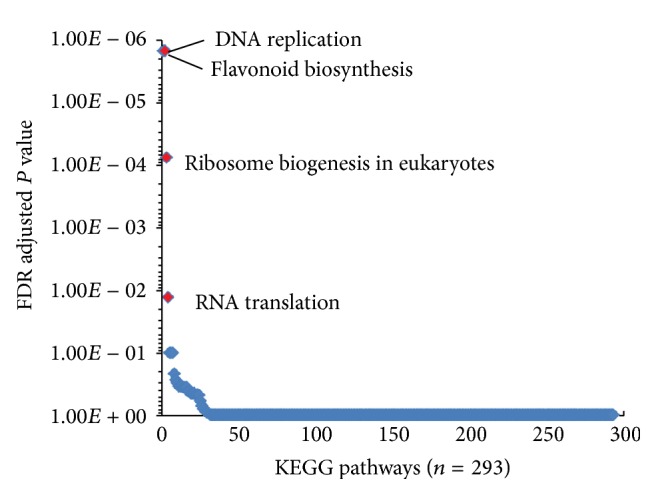
Pathway enrichment based on the profile of combined unique gene expression.

**Figure 5:**
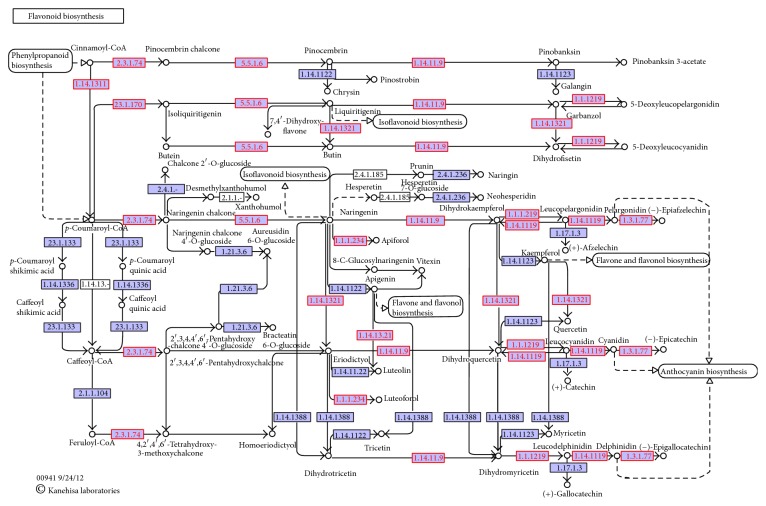
Genes encoding enzyme commission (ECs) in flavonoid biosynthesis pathway were upregulated (red) in the transcriptome of resistant crape myrtle genotype (“Natchez,” NAT) compared to susceptible crape myrtle genotype (“Carolina Beauty,” CAB) transcriptome.

The observation of significant upregulation of KEGG genes in secondary metabolism pathways (e.g., benzoxazinoid and flavonoid biosynthesis) prompted our interest to examine whether these potential plant defense-related pathways are more abundant in the resistant genotype. To address this question, a Fisher's exact test was performed. As a result, only 4 out of ~300 KEGG pathways showed significant difference between resistant and susceptible genotypes (Figure 4). Moreover, flavonoid biosynthesis pathway (ko00941) was the most significantly enriched pathway (Figure 4). In addition, 11 out of 19 KEGG genes in flavonoid biosynthesis pathway have transcripts assembled, and 7 out of 11 were significantly upregulated in the resistant genotype as shown in Figure 5.

### 3.5. Comparison of Transcripts Abundance of Potential Trait Associated Genes

**Figure 6:**
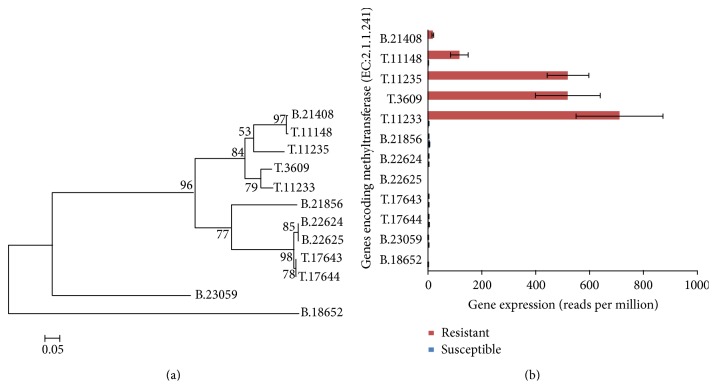
(a) Phylogenetic distance of genes encoding methyltransferases to produce DIMBOA-glucoside. (b) Comparison of transcripts abundance (reads per million, RPM) assembled unigenes encoding methyltransferases.

To specifically identify the assembled genes that are potentially linked with the trait of powdery mildew resistance, the top two differentially expressed KEGG genes (K13230 and K05280) were described in detail. KEGG gene K13230 encodes for 2,4,7-trihydroxy-1,4-benzoxazin-3-one-glucoside 7-O-methyltransferase, an enzyme involved in the biosynthesis of the protective and allelopathic benzoxazinoid DIMBOA, which serves as a natural defense against a wide range of pests including insects, pathogenic fungi, and bacteria [32, 33]. There are a total of 12 assembled unigenes (alleles) that were annotated as K13230. Cluster analysis of the 12 assembled unigenes based on their sequence similarity indicates that these unigenes group into three clusters (Figure 6(a)). To identify which clusters of unigenes have significant upregulation, the transcriptome reads from resistant and susceptible genotypes were aligned onto the 12 assembled unigenes and found the cluster with unigenes. T.11233, T.3609, T.11235, T.11148, and B.21408 show almost no expression in the susceptible genotype but have up to ~800 RPM transcripts abundance in resistant genotype (Figure 6(b)).

**Figure 7:**
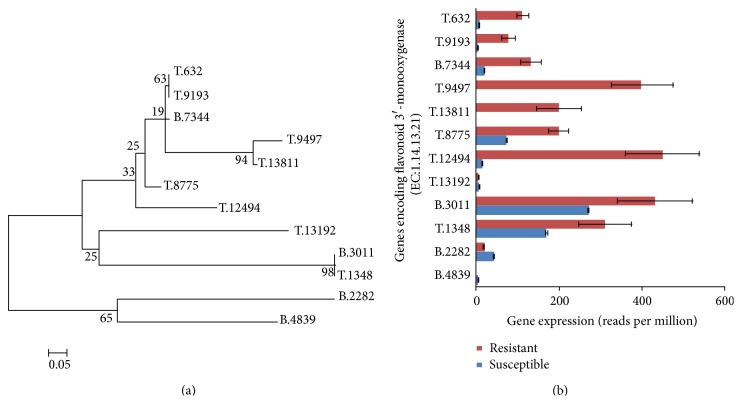
(a) Phylogenetic distance of unigenes encoding flavonoid 3′-monooxygenase to produce flavonoids. (b) Comparison of transcripts abundance (reads per million, RPM) assembled unigenes encoding flavonoid 3′-monooxygenase.

Phylogenetic analysis and comparison for KEGG gene encoding flavonoid 3′-monooxygenase involved in the reaction to produce flavonoid (e.g., Quercetin) were performed. Flavonoids are a major class of plant secondary metabolites that serve a multitude of functions including plant pigment for flower coloration, inhibitory activity against organisms that cause plant disease, antibacterial activity, synergistic activity with antibiotics, ability to suppress bacterial virulence factors, and antioxidant activity [34, 35]. A total of 12 assembled unigenes were retrieved and assigned with the function of flavonoid 3′-monooxygenase. Phylogenetic analysis divided them into two groups (Figure 7) and 10 out of 12 unigenes have significant upregulation of transcripts in NAT genotype even though NAT has white flowers.

### 3.6. Validation of Illumina Expression Patterns by qRT-PCR

To confirm the transcript abundance differences identified by the redundancies of the transcriptome reads per million (RPM) in resistant genotype NAT, 16 candidate genes, nine overexpressed gene sequences in CAB genome and seven from NAT genome, were selected and their expression was detected by qRT-PCR. The fold increase was calculated by treating CAB as the treatment and NAT as the control. For the NAT primers, which were designed from the overexpressed gene sequences in NAT genome, NAT was used as the treatment and CAB as the control. Three housekeeping genes (cab-h1, cab-h8, and nat-h1) were run together in the qRT-PCR reactions (Table 3). The results indicated that six genes (corresponding primers cab-rt1, cab-rt2, cab-rt5, cab-rt7, cab-rt8, and cab-rt9) are highly expressed in cultivar CAB, and three genes (corresponding primers nat-rt8, nat-rt-11, and nat-rt14) are highly expressed in cultivars NAT. Based on the analysis of the RPM in these two genotype genomes, these selected highly expressed genes or alleles are highly expressed in genotype NAT or CAB. The deviation of the expression validated in RT-PCR is unknown because the samples of this study did not include technical replications instead of biological replications.

## 4. Discussion

Of the 56 [3] to 80 [4] species in genus* Lagerstroemia*, the most widely planted species, known as crape myrtle, are* L. indica* and* L. indica* ×* L. fauriei* hybrids [1]. Because of long juvenile growth, tree breeding is lagged behind other crops. In recent years, the newly developed NGS-based RNA-seq technique has been widely used for transcriptome sequencing and* de novo* assembly, discovery of novel genes, and investigation of gene expression in many nonmodel trees such as bamboo [36], coffee [37], eucalyptus tree [38, 39], oak [40], pine [41], and poplar [42, 43]. Because the genome sequence of the genus* Lagerstroemia* is still not available, transcriptome sequence (RNA-seq) analysis has become one of the most efficient ways to discover and identify novel genes of interests. In this study, based on transcriptome sequencing and* de novo* assembly, a total of 224,443 assembled transcripts including 37,035 unigenes were obtained from leaf cDNA libraries of powdery mildew resistant NAT and susceptible CAB* L. indica* cultivars. These unigenes may or may not be involved in the plant defense against powdery mildew in* Lagerstroemia* species due to the limited experiment design, that is, no inoculated and inoculated genotype comparison, or in flower coloration. However, we may assume the potentials to these important traits (disease resistance and flower coloration) based on the genotypes selected in this study.

A total of 23,564 and 23,387 (37,035 when combined) unigenes were identified in resistant NAT and susceptible CAB genome, respectively. Given the genetic background at the gene function, or transcript level, most transcripts were annotated by BLAST and functional bioinformatics analyses, indicating that the sequences of transcripts were assembled and annotated correctly. However, there is still an abundance of unknotted transcripts that remained as singletons in the unigene set (data not shown), which showed no hits against NCBI nr database. These singletons could result from sequencing error, restrictions of assembler algorithm being used in the calculation program [44], and/or other factors [45]. It was suggested that greater than 40 million reads should be sufficient to identify functional genes [46]. In this study, nearly half of the transcriptome sequences were duplicated (48.3% in CAB and 47.1% in NAT, resp.). A relatively small portion of reads (~34.6 million) were assembled into contigs and provided a starting point to discover candidate genes. In fact, many unassembled reads or singletons were high quality reads and matched to proteins in BLAST searches in the NCBI databases, suggesting that they are still an important source of information and most likely are rare genes with very low expression level. To find out more functional genes, more deep sequencing will be needed for further analyses.

Of these unigenes, 475 differentially expressed genes (DEGs) were identified in genus* Lagerstroemia* genome. Some of the DEGs have been validated by RT-PCR. However, it is necessary to further confirm these gene functions with regard to disease resistance or flower coloration in* Lagerstroemia* species. Using the top 10 differentially expressed KEGG genes between susceptible CAB and resistant NAT crape myrtle cultivars, five KEGG genes were found to be involved in the biosynthesis of secondary metabolites. Among them, 2,4,7-trihydroxy-1,4-benzoxazin-3-one-glucoside 7-O-methyltransferase (K13230), a gene encoding enzymes [EC:2.1.1.241] involve in the last step of benzoxazinoid biosynthesis to produce DIMBOA-glucoside, has 67.7-fold difference in the resistant cultivar when compared to the susceptible cultivar. Benzoxazinoids are secondary metabolites that are effective in defense and allelopathy and therefore play a role in plant defense against herbivorous insects and pathogens [33]. In addition, four of the top 10 KEGG genes came from flavonoid biosynthesis pathway (ko00941). Flavonoids are widely distributed in plants and the most important plant pigments for flower coloration. It is not surprising that genes involved in the flavonoid biosynthesis are differentially expressed between these two cultivars because NAT produces white flowers and CAB blooms are red. In addition, some flavonoids have inhibitory activity against organisms that cause plant diseases, such as* Fusarium oxysporum* [35]. Although there was no artificial inoculation with identified (or purified) inoculum sources of* E. australiana*, infected foliage of other* L. indica* plants was used as inoculum before leaf collection for cDNA construction. In addition, this study did not set up infected and noninfected experiments for each genotype. Therefore, all highly expressed transcripts could not be effectively linked with either powdery mildew or flower color traits, although the top two genes are identified to be involved in plant defense or flower coloration biosynthesis pathway. However, the fact that certain differences in transcript abundance have been found between these two genotypes kindles our interests in further investigating the true linkage between expression level and disease resistance or flower color trait. On-going projects to sequence the F1 segregating population of the cross CAB × NAT will discover the key genes that are associated with the segregation of powdery mildew resistance in* L. indica* species. Transcriptome data presented here will provide a comprehensive understanding of the gene transcription profiles of crape myrtle and lay a solid foundation for further investigation of plant defense against powdery mildew and identify novel gene(s) in this species. The transcriptome analyses provide an efficient starting point for characterizing functional genetic variation in nonmodel organisms, especially woody ornamental plants.

## 5. Conclusions

The transcriptome sequences in leaves of two* Lagerstroemia indica* cultivars were first reported in this study. A total of 224,443 assembled transcripts representing 37,035 unigenes in both genotypes were obtained. Based on the assembled* de novo* transcriptome, 475 differentially expressed genes (DEGs) were found to differ significantly between genotypes using four different statistic methods. Of the top ten DEGs, five were related to plant defense and four related to antioxidant activities. Five in benzoxazinoid biosynthesis pathway and seven unigenes in flavonoid biosynthesis pathway were significantly upregulated in the resistant cultivar. The expression patterns of selected DEGs were further validated with qRT-PCR, which showed general agreement with the abundances of the assembled transcripts in resistant or white flower trait. The highly expressed transcripts are a useful resource to discover candidate genes that have potential association with either powdery mildew resistance or flower coloration in genus* Lagerstroemia*. The molecular basis of the response to powdery mildew stress in* Lagerstroemia* species was preliminarily characterized in this study, which resulted in useful information and laid a solid foundation for further investigating the molecular regulation mechanism of powdery mildew resistance in woody ornamental plants.

## Supplementary Material

Supplement Table S1. Primer sequence, annealing temperature and expected size from differentially expressed genes sequences.

## Figures and Tables

**Table 1:** Summary of sequencing data of two *Lagerstroemia indica* cultivars: powdery mildew susceptible (“Carolina Beauty,” CAB) and powdery mildew resistant (“Natchez,” NAT), resulting from Illumina deep sequencing.

	CAB	NAT	Combined
Total sequence bases	1,765,922,760	1,874,976,180	3,640,898,940
Total reads	16,818,312	17,856,916	34,675,228
Average length of raw read (bp)	105	105	
Number of contigs	111,804	112,639	224,443
Average length of contigs (bp)	671	581	626
Total length of all contigs (bp)	64,344,103	64,421,335	128,765,438
N50 contig size (bp)	1110	1081	
Minimum length of contig (bp)	145	142	
Maximum length of contig (bp)	11,309	7,412	
GC percentage (%)	47.57	47.72	
Number of unique genes	23,654	23,387	37,035
Average length of unigenes (bp)	878	860	835
Annotated transcripts (% of unigenes)	20118 (85.05%)	19898 (85.08%)	

**Table 2:** Top 10 differentially expressed KEGG genes between powdery mildew susceptible (“Carolina Beauty,”  CAB) and powdery mildew resistant (“Natchez,”  NAT) crape myrtle genotypes.

KEGG ID	KEGG pathway ID	Pathway	Definition	Fold change	*P* value		
					EdgeR	DESeq	Rankprod
K13230	ko00402	Benzoxazinoid biosynthesis	2,4,7-Trihydroxy-1,4-benzoxazin-3-one-glucoside 7-O-methyltransferase [EC:2.1.1.241]	67.7	0.00*E* + 00	6.89*E* − 30	0
K05280	ko00941	Flavonoid biosynthesis	Flavonoid 3′-monooxygenase [EC:1.14.13.21]	3.49	4.80*E* − 76	1.03*E* − 07	0.00068
K00660	ko00941	Flavonoid biosynthesis	Chalcone synthase [EC:2.3.1.74]	2.28	3.83*E* − 44	5.79*E* − 08	0.00472
K00517				1.7	3.33*E* − 19	0.000124	0.0288
K00487	ko00941	Flavonoid biosynthesis	*trans*-Cinnamate 4-monooxygenase [EC:1.14.13.11]	3.31	2.04*E* − 68	5.20*E* − 07	0.000766
K11251	ko05034	Alcoholism	Histone H2A	1.59	6.31*E* − 22	6.94*E* − 21	0.041
K11254	ko05035	Alcoholism	Histone H4	1.57	5.60*E* − 21	6.81*E* − 20	0.0464
K08081	ko00960	Tropane, piperidine, and pyridine alkaloid biosynthesis	Tropine dehydrogenase [EC:1.1.1.206]	7.37	0.00*E* + 00	4.63*E* − 251	8.10*E* − 05
K00549	ko00270	Cysteine and methionine metabolism	5-Methyltetrahydropteroyltriglutamate–homocysteine methyltransferase [EC:2.1.1.14]	1.65	1.74*E* − 17	0.000147	0.0351
K10775	ko00360	Phenylalanine metabolism	Phenylalanine ammonia-lyase [EC:4.3.1.24]	1.99	1.18*E* − 19	0.00886	0.0133
K00276	ko00260; ko00350; ko00360; ko00410; ko00950; ko00960		Primary-amine oxidase [EC:1.4.3.21]	3.91	2.47*E* − 86	4.64*E* − 08	0.000429
K01583	ko00330	Arginine and proline metabolism	Arginine decarboxylase [EC:4.1.1.19]	8.82	1.12*E* − 181	3.34*E* − 12	5.52*E* − 05
K01904	ko00130; ko00360; ko00940		4-Coumarate–CoA ligase [EC:6.2.1.12]	2.04	3.35*E* − 31	1.23*E* − 05	0.00942
K03327			Multidrug resistance protein, MATE family	1.56	1.79*E* − 17	1.39*E* − 09	0.0493
K01890	ko00970	Aminoacyl-tRNA biosynthesis	Phenylalanyl-tRNA synthetase beta chain [EC:6.1.1.20]	2.83	7.19*E* − 95	1.01*E* − 38	1.56*E* − 03
K06568				1.66	1.27*E* − 23	1.10*E* − 11	0.0319
K05279	ko00944	Flavonol 3-O-methyltransferase	Flavonol 3-O-methyltransferase [EC:2.1.1.76]	2.33	1.59*E* − 68	9.41*E* − 58	0.00373

**Table 3:** Relative quantification and fold expression of differentially expressed genes in powdery mildew resistant “Natchez” (NAT) versus susceptible “Carolina Beauty” (CAB) cultivars.

Primer	Query name	NAT cDNA Ct (mean)	CAB cDNA Ct (mean)	Fold diff. (2^-Nat-CabCt^)^*∗*^	Fold diff. (2^-Cab-NatCt^)^*∗∗*^	Fold diff. (2^-Nat-CabCt^)^*∗*^	Fold diff. (2^-Cab-NatCt^)^*∗∗*^	Fold diff. (2^-Nat-CabCt^)^*∗*^	Fold diff. (2^-Cab-NatCt^)^*∗∗*^	Mean fold
**cab-h1**	**B.9985**	**27.8**	**26.78**							
*cab-h8 *	*B.8346 *	*27.83 *	*28.75 *							
***nat-h1***	***T.9454***	***29.40***	***29.30***							
cab-rt1	B.7344	32.18	30.94	—	**1.16**	—	*4.47 *	—	***2.20***	2.61
cab-rt2	B.2282	30.19	29.92	—	**0.59**	—	*2.28 *	—	***1.13***	1.33
cab-rt3	B.3011	31.50	33.31	—	**0.14**	—	*0.54 *	—	***0.27***	0.32
cab-rt5	B.18652	29.02	26.48	—	**2.87**	—	*11.00 *	—	***5.43***	6.43
cab-rt6	B.21408	35.94	37.44	—	**0.17**	—	*0.67 *	—	***0.33***	0.39
cab-rt7	B.21856	35.16	33.60	—	**1.45**	—	*5.58 *	—	***2.75***	3.26
cab-rt8	B.22624	33.80	28.64	—	**17.63**	—	*67.65 *	—	***0.17***	28.48
cab-rt9	B.22625	29.36	26.84	—	**2.83**	—	*10.85 *	—	***5.35***	6.34
cab-rt10	B.23059	32.30	34.69	—	**0.09**	—	*0.36 *	—	***0.18***	0.21
nat-rt6	T.13188	32.75	37.73	**0.20**	—	*0.05 *	—	***0.11***	—	0.12
nat-rt8	T.13192	28.34	29.43	**4.23**	—	*1.10 *	—	***2.23***	—	2.52
nat-rt9	T.13486	32.08	29.40	**0.21**	—	*0.05 *	—	***0.11***	—	0.12
nat-rt11	T.17520	37.91	28.78	**1.79**	—	*0.47 *	—	***0.95***	—	1.07
nat-rt13	T.8775	28.14	27.46	**1.27**	—	*0.33 *	—	***0.67***	—	0.75
nat-rt14	T.9193	29.82	31.05	**4.76**	—	*1.24 *	—	***2.51***	—	2.84
nat-rt15	T.17643	30.33	28.44	**0.55**	—	*0.14 *	—	***0.27***	—	0.32

*Note*. We calculated the fold increase in expression for the NAT primers by treating NAT as the treatment and housekeeping gene as the control and compared to CAB treatment (*∗*). For the CAB primers we used CAB as the treatment and housekeeping gene as the control and compared to NAT treatment (*∗∗*). Highlight in bold (cab-h1), italic (cab-h8), and bold italic (nat-h1) indicates that fold difference of expression was calculated based on individual housekeeping gene. Mean fold was calculated from three housekeeping genes.
